# Understanding the Time‐Dependent Mechanical Behavior of Bimodal Nanoporous Si–Mg Films via Nanoindentation

**DOI:** 10.1002/gch2.201800100

**Published:** 2019-02-12

**Authors:** Tyler L. Maxwell, Thomas John Balk

**Affiliations:** ^1^ Department of Chemical and Materials Engineering University of Kentucky 177 F. Paul Anderson Tower Lexington KY 40506‐0046 USA

**Keywords:** mechanical behavior, nanoindentation, porous, thin films

## Abstract

This study addresses the mechanical response of nanoporous Si–Mg films, which are fabricated using free‐corrosion dealloying and which represent an intriguing form of silicon that may find use as an anode material in lithium‐ion batteries. The porous thin‐film samples, in both the as‐dealloyed and annealed states, are designed to have a final thickness of ≈1 µm so that substrate effects can be avoided during mechanical characterization in both the time and frequency domains. The as‐dealloyed and annealed samples are investigated using a modified continuous stiffness measurement (CSM) technique that optimizes the ability to achieve steady‐state harmonic motion, such that accurate phase angle measurements can be obtained; the as‐dealloyed and annealed samples exhibit distinct phase angles of 1.9° and 2.6°, respectively. Observations made in the time domain suggest that the time dependence of nanoporous Si–Mg stems largely from plasticity. The reduced modulus values of as‐dealloyed and annealed samples are investigated using the CSM technique and have corresponding values of 5.78 and 11.9 GPa, respectively. Similarly, the hardness of as‐dealloyed and annealed samples are 167 and 250 MPa, respectively.

Nanoindentation is an efficient method for probing small volumes of material, with dimensions on the order of 1 µm, to obtain mechanical properties that can inform engineering applications.^1^, ^2^, ^3^ Since the inception of the first commercially available indentation apparatus in the 1980s, progress has been rapid and the technique has evolved significantly, with a notable milestone being the advent of the continuous stiffness measurement (CSM) technique and the additional changes that stemmed from it.^3^, ^4^ The continuous improvement that has occurred over the last few decades has largely resulted from experimental research on materials that are well‐behaved elastic–plastic solids, e.g., fused silica. Thin films of porous materials that exhibit time dependence are among the most challenging materials to characterize with nanoindentation, but strategic modification of indentation techniques and careful interpretation of data can facilitate the extraction of pertinent mechanical behavior.^3^, ^5^, ^6^, ^7^, ^8^, ^9^, ^10^, ^11^, ^12^

The motivation for this study comes from a combination of preliminary data observed by Jiang et al. and from lithium‐ion battery research.^13^, ^14^, ^15^, ^16^, ^17^ While silicon is an attractive anode material for lithium‐ion batteries due to its high charge capacity, it also suffers from cracking and pulverization during cycling.^18^ By increasing the surface‐area‐to‐volume ratio, it is possible for silicon to undergo a brittle‐to‐ductile transition.^19^ Creating nanoporous silicon (np Si) is an effective method to increase the surface‐area‐to‐volume ratio and may mitigate brittle failure. Jiang et al. performed in situ nanoindentation of np Si films ≈100 nm thickness in the transmission electron microscope.^15^ These films were produced in a similar manner to those made by Maxwell et al., which are the subject of the current study.^15^, ^20^ The results from Jiang et al. indicate that np Si can recover large deformations after indentation, but that study was performed on films that are too thin to be accurately characterized with nanoindentation. As such, the thicker nanoporous silicon–magnesium (np Si–Mg) films produced by Maxwell et al. are better suited to nanoindentation.^20^ In the current study, a modified CSM technique was used to investigate the damping characteristics of np Si–Mg films in the frequency domain via phase angle measurements.^8^, ^11^ These results were interpreted alongside data collected in the time domain, to facilitate modeling the deformation behavior of np Si–Mg films.

The modified CSM technique used here was first described by Herbert et al., and leverages the ability to achieve steady‐state harmonic motion—a necessary condition to accurately determine specimen damping.^8^ Additionally, the specimen must dominate the measured phase angle response. This condition exists when Equations (1) and (2) are satisfied. Finally, measurements to correct the reference phase angle are established using Equations (3)–(5), to account for shifts in the displacement electronics.^8^, ^10^ The close agreement between measured and corrected phase angle values of well‐known materials (polymethyl methacrylate, PMMA; and fused silica) were interpreted as corroboration of experimental accuracy.

(1) $${\frac{f_{0}}{h_{0}}|}_{\mathrm{coupled}}\gg \;\;{\frac{f_{0}}{h_{0}}|}_{\mathrm{free}\;\mathrm{space}}$$

(2) $$K_{\mathrm{lf}}\gg K_{\mathrm{contact}}$$

(3) $$\;\delta_{\mathrm{corrected}}=\;\;\tan^{-1}\left(\frac{C_{\mathrm{contact}}\omega }{K_{\mathrm{contact}}-\;\;m\omega^{2}}\right)\;\;$$

(4) $$C_{\mathrm{contact}}\omega \;=\;\;{\frac{f_{0}}{h_{0}}\sin\delta |}_{\mathrm{coupled}}\;-\;\;{\frac{f_{0}}{h_{0}}\sin\delta |}_{\mathrm{free}\;\mathrm{space}\;}$$

(5) $$K_{\mathrm{contact}}-\;\;m\;\omega^{2}={\left[\frac{1}{{\frac{f_{0}}{h_{0}}\cos\delta |}_{\mathrm{coupled}}-\;\;{\frac{f_{0}}{h_{0}}\cos\delta |}_{\mathrm{free}\;\mathrm{space}}}\;\;-\;\;\frac{1}{K_{\mathrm{lf}}}\right]}^{-1}\;$$

where (*f*_0_/*h*_0_)|_coupled_ is the apparent power of the coupled actuator and specimen, (*f*_0_/*h*_0_)|_free space_ the apparent power of the actuator hanging in free space, *K*_lf_ the load frame stiffness (taken to be 1.59 × 10^6^ N m^−1^),^21^ *K*_contact_ the contact stiffness between the indenter tip and sample, δ_corrected_ the phase angle of the sample, *C*_contact_ω^2^ the lag, and (*K*_contact_ – *mω* ^2^) the in‐phase components of the imposed oscillation required for the CSM technique. This summarizes the framework that underpins the analysis of phase angle data obtained for np Si–Mg thin film samples.

The validation experiments, shown in **Figure** **1**, were performed on fused silica and PMMA because both materials exhibit well‐known phase angle behavior. The true phase angle values for fused silica and PMMA, when measured at room temperature and 45 Hz, are quoted in the literature as 0° and 3.4°, respectively.^8^, ^22^, ^23^ Each phase angle value falls well within the range of one standard deviation about the average experimental phase angle at 45 Hz. Moreover, measured and corrected values are nearly identical when plotted as a function of frequency, indicating the reference phase angle is accurate. This demonstrates that the modified CSM technique can achieve steady‐state harmonic motion, that the measured phase angle is not influenced by shifts resulting from the displacement electronics, and that the phase angle of the specimen dominates the response. It is also observed in Figure 1 that the average phase angle value for fused silica is closer to the true value at frequencies higher than 45 Hz. This likely results from the nanoindenter's ability to better achieve steady‐state harmonic motion when the change in contact area is insignificant over the time scale of the measurement.^8^ For this reason, the results for np Si–Mg thin films will be reported at a frequency of 70 Hz in this article.

**Figure 1:**
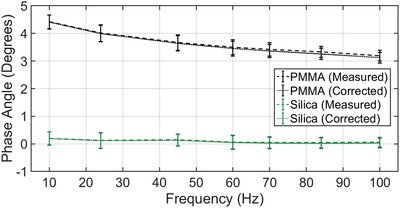
Proof‐of‐concept plot showing measured and corrected values for both a known linear elastic solid (fused silica) and a known linear viscoelastic solid (PMMA). This demonstrates that the reference phase calibration built into the software accounts for shifts associated with displacement electronics.

Representative load–depth curves of PMMA, silica, and np Si–Mg are shown in **Figure** **2**. The key features of these plots are circled at the summit and base of the curves. The plateau near the top of each load–depth curve represents material creep during a 60 s hold period. Of course, material creep is convoluted with transient drift effects.^11^ Instrument drift was measured after nearly complete unloading, denoted by a circled region near the base of each curve. The extent to which the known viscoelastic solid PMMA creeps during the 60 s hold period is ≈22 nm, and a similar 22 nm recovery is also observed at the base of the curve after unloading (Figure 2a). It is necessary to compare the values obtained on PMMA to those for the known linear elastic solid fused silica, to determine how much of the 22 nm displacement can be attributed to transient drift effects, which in this case is 3–5 nm (as indicated by the circled region near the base of the curve in Figure 2b). Similarly, np Si–Mg sample creep was ≈11 nm during the 60 s hold period (Figure 2c), and transient drift was assumed to account for 3–5 nm. These experiments were performed under identical conditions and indicate that np Si–Mg and PMMA exhibit some level of time dependence, unlike fused silica. Moreover, PMMA appears to have a slightly greater time dependence than np Si–Mg. It is noted that PMMA recovers almost the entire 22 nm displacement (from the creep period) during subsequent measurement of drift near the base of the curve, whereas np Si–Mg shows negligible recovery. Since PMMA is a known viscoelastic solid, it stands to reason that it would recover fully at low loads. The observation that np Si–Mg does not exhibit significant recovery implies that its time dependence stems largely from plasticity. Therefore, an appropriate model to describe the mechanical behavior of np Si–Mg should incorporate both time‐dependent plasticity and viscoelasticity, as in the viscous–elastic–plastic (VEP) model developed by Oyen.^24^, ^25^, ^26^ However, further investigation would be required to understand the mechanism(s) of deformation.

**Figure 2:**
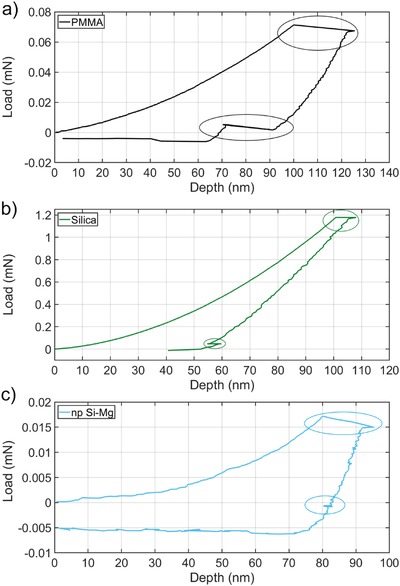
Mechanical response of reference materials and np Si–Mg, measured in the time domain. The response of a) PMMA is compared to that of b) fused silica and c) np Si–Mg at similar depths and under the same experimental conditions, such that transient effects are comparable between tests. PMMA and np Si–Mg exhibit time dependence, while fused silica does not.

There are two domains in which a nanoindentation experiment can be performed, namely the time domain and the frequency domain, each with inherent advantages and disadvantages.^11^ Thermal drift is a major obstacle to testing in the time domain—as in the case of creep. However, a significant advantage of operating in the frequency domain is that sample stiffness and damping are unaffected by transient effects—as in the case of the modified CSM technique. As such, the phase angle values reported in **Table** **1** are true quantitative measurements of damping capacity for the materials tested. The quantitative results in Table 1 corroborate the qualitative results in Figure 2, and indicate that PMMA exhibits more damping than np Si–Mg.

**Table 1:** Relevant values in the calculation of energy dissipated by the materials tested at a frequency of 70 Hz. The damping capacity of np Si–Mg lies between that of fused silica and PMMA

	Fused silica	PMMA	As‐dealloyed np Si–Mg	Dealloyed/annealed np Si–Mg
$\frac{\frac{f_{0}}{h_{0}}{|}_{\mathrm{coupled}}}{\frac{f_{0}}{h_{0}}{|}_{\mathrm{free}\;\mathrm{space}}}$	1000	453	23.7	25.5
$\frac{K_{\mathrm{lf}}}{K_{\mathrm{contact}}}$	9.81	22.9	552	571
δ ± σ	0.0 ± 0.2	3.3 ± 0.2	1.9 ± 0.5	2.6 ± 1.4
$\frac{\left(\delta_{\mathrm{measured}}-\delta_{\mathrm{corrected}}\right)}{\frac{1}{2}(\delta_{\mathrm{measured}}+\delta_{\mathrm{corrected}})}$	≈0%	1.8%	36%	18%

The ratios of ${\frac{f_{0}}{h_{0}}|}_{\mathrm{coupled}}$to ${\frac{f_{0}}{h_{0}}|}_{\mathrm{free}\;\mathrm{space}}$ and *K*_lf_ to *K*_contact_ are shown in Table 1 for the np Si–Mg tested in this study, and show that Equations (1) and (2) hold true in each case. These conditions must be satisfied if the instrument's contribution to the measured phase angle is to be neglected.^8^ The percent difference between δ_measured_ and δ_corrected_ is also shown in Table 1. The results for PMMA and fused silica support the results shown in Figure 1. There is a larger percentage difference between the measured and corrected values for the np Si–Mg samples because the depth (and consequently the contact area) at which the phase angle measurements were obtained are smaller. This underscores the importance of correcting for shifts in displacement electronics as the contact dimensions approach zero and Equations (3)–(5) are used.^8^

Representative load–displacement curves, as well as plots of reduced modulus (*E*_r_) and hardness of the np Si–Mg films as a function of indentation depth, are shown in **Figure** **3**. It is known that modulus and hardness data obtained on materials that exhibit time dependence are subject to error due to pile‐up around the indenter.^3^ Conveniently, there exists a readily obtained experimental parameter that can be used to estimate the extent to which pile‐up affects the contact area, namely the ratio *h*_f_/*h*_max_.^3^ Here, *h*_f_ represents the final indentation depth and *h*_max_ represents the depth at peak load. A material with *h*_f_/*h*_max_ ratio near unity exhibits completely plastic behavior, while materials with a ratio near zero are fully elastic. In terms of contact area, however, materials with a ratio >0.7 could be subject to underestimating the contact area due to pile‐up. The load–displacement curves in Figure 3a indicate *h*_f_/*h*_max_ ratios of 0.80 and 0.86 for as‐dealloyed and annealed np Si–Mg, respectively. Depending on the amount of work‐hardening, the contact area is likely underestimated slightly. This indicates that the modulus and hardness values reported in Figure 3b,c could be somewhat overestimated. It should be noted, however, that this ratio is normally applied to solid materials, i.e., nonporous. The mechanism leading to an inflated ratio is typically explained as pile‐up around the indenter tip, in the context of solid materials. For porous materials, however, the mechanism would more likely be densification than pile‐up, as this is a well‐documented phenomenon to occur during indentation of porous materials.^27^, ^28^, ^29^, ^30^, ^31^ Nevertheless, the effect on modulus and hardness is the same as if the contact area were underestimated; modulus and hardness are overestimated to some degree.

**Figure 3:**
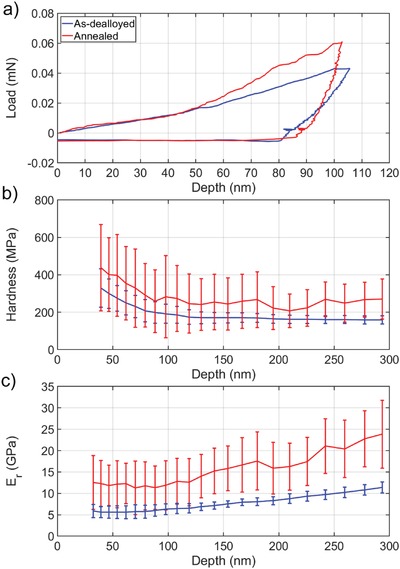
The load–displacement curves in (a) show that both the as‐dealloyed (blue) and annealed (red) films exhibit significant plasticity as determined by the *h*_f_/*h*_max_ ratio. The hardness in (b) and the reduced modulus in (c) were obtained using the CSM technique. There is a plateau in the reduced modulus at depths less than 100 nm, which reflects the elastic modulus of the np Si–Mg films. Beyond 100 nm indentation depth, the reduced modulus values increase due to the influence of the silicon substrate. The hardness plots exhibit plateaus at depths greater than 100 nm, which is reasonable for a soft film on a hard substrate.

There is a well‐defined plateau in *E*_r_ values for both as‐dealloyed and annealed np Si–Mg films, but there is greater variability in *E*_r_ of the annealed sample. Average values for *E*_r_ are 5.78 and 11.87 GPa for as‐dealloyed and annealed np Si–Mg, respectively. Typically, the modulus of the film dominates the elastic response of the film–substrate system at depths less than 10% of the film thickness, corresponding to ≈100 nm in this case, after which the modulus approaches that of the substrate.^29^, ^30^ This is reflected here by a gradual increase in modulus at depths larger than 100 nm, as shown in Figure 3b. The microstructural features of np Si–Mg films were described previously by Maxwell and Balk and show that nanocrystals form after vacuum annealing.^20^ This could make annealed samples more heterogeneous throughout the film thickness and explain the higher variability of *E*_r_. Moreover, these nanocrystals may act to increase the average stiffness response on unloading.^32^ The increased modulus of the annealed film is not the result of film densification due to annealing. The as‐dealloyed film does contract by ≈11% during annealing, which corresponds to an increase in relative density and influences the modulus, e.g., per the Gibson and Ashby relation.^33^, ^34^ The increase in modulus that would result from an increase in relative density is significantly lower than the measured increase in reduced modulus. This modulus increase therefore appears to reflect a change in material property, rather than resulting from the slight increase in relative density. Moreover, if the film had contracted significantly, the onset of a substrate effect for the annealed film should appear at a much lower depth than that observed for the as‐dealloyed film. However, this was not the case, and instead the hardness values of the two samples each exhibit a plateau starting at an indentation depth ≈100 nm. The hardness values measured here are 167 and 250 MPa for as‐dealloyed and annealed np Si–Mg, respectively. Hardness measurements of soft films on hard substrates are accurate at depths up to 50% of the film thickness.^29^, ^30^ The hardness values here are accurate and constant up to the point of maximum indentation depth (300 nm), which is less than half the film thickness.

The mechanical behavior of np Si–Mg films with thickness of ≈1 µm, fabricated via free‐corrosion dealloying, were investigated using nanoindentation in both the time and frequency domains, and these results may inform future advances in the design of anode materials for lithium‐ion battery applications. The np Si–Mg exhibited time dependence when tested in the time domain; a well‐known viscoelastic material (PMMA) and a well‐known linear elastic material (fused silica) were used as references. The limited recovery of np Si–Mg films after nearly complete unloading indicates that its time dependence can be primarily attributed to plastic deformation. A modified CSM technique was used to quantitatively characterize the phase angle of as‐dealloyed and annealed np Si–Mg films, with measured phase angle values of 1.9° and 2.6°, respectively. The reduced elastic modulus was evaluated for as‐dealloyed and annealed films using the CSM technique, with values of 5.78 and 11.9 GPa, respectively. The hardness of the as‐dealloyed and annealed films were 167 and 250 MPa, respectively.

## Experimental Section

*Film Fabrication*: All samples were dealloyed in distilled water as described in an earlier publication where the final thickness was nominally 1 µm.^20^ Annealing of samples was performed in vacuum, also in the same manner presented in that article. The microstructure was verified by scanning electron microscopy (SEM) in cross section and plan view, and all samples tested in the current study exhibited a thin surface layer.

*Sample Preparation*: Nanoindentation experiments were performed with an iMicro load frame and an iNano actuator (Nanomechanics, Oak Ridge, TN). This combination was chosen because the iMicro load frame offers a larger associated stiffness than the iNano, and the iNano actuator offers better sensitivity at small depths. Standard samples of fused silica and PMMA were tested, to validate phase angle measurements and showed that the dynamic response was dominated by the specimen. Fused silica is a well characterized elastic–plastic solid and was used to calibrate the tip area function of the diamond Berkovich indenter (Microstar Tech, Huntsville, TX).^8^ The silica specimen (12.9 × 12.9 × 3.5 mm) was a standard reference block provided by Nanomechanics, whereas the PMMA (cut to size: 14.5 × 14.5 × 6.1 mm) was purchased from McMaster Carr. The silica arrived attached to an aluminum puck, whereas the PMMA and other samples used for indentation were attached to aluminum pucks with a thin layer of mounting adhesive (Crystal Bond; SPI Supplies, West Chester, PA). The samples were pressed carefully, to ensure excess air and adhesive were removed from the sample while drying. Sixteen indents were performed on a specimen in a 4 × 4 array, where the distance between adjacent indents was at least 30 times the maximum indentation depth.

*Nanoindentation Testing*: Samples with a nominal thickness of 1 µm were strategically fabricated to safely ignore substrate effects when indenting up to 10% of the film thickness (100 nm) while using a Berkovich tip.^29^, ^35^ In the modified CSM technique used in this study, the indenter hold period began after it reached a depth of 80 nm. It was determined that a hold period of 60 s was sufficient for the sample's response to the dynamic oscillation of 2 nm to reach steady state. However, only data from the last 5 s of the 60 s hold period were averaged at each frequency. It should be noted, however, that the data in Figure 1 were collected at ≈2400 and 600 nm indentation depth for PMMA and fused silica, respectively. A large depth was used for PMMA because it has been documented that a processing effect can give rise to an overestimation of phase angle value at depths below ≈1500 nm, and a similar phenomenon was observed in the current study.^8^ The authors are aware that instrument compliance and damping in the actuator can be exacerbated at small contact depths. The instrument used in this study to investigate damping for small contact areas used a state‐of‐the‐art phase‐lock amplifier designed to test materials at low loads and depths.

## Conflict of Interest

The authors declare no conflict of interest.
